# Nitric Oxide in the Treatment of COVID-19: Nasal Sprays, Inhalants and Nanoparticles

**DOI:** 10.1155/bri/8846903

**Published:** 2025-10-12

**Authors:** Amarley Wright, Donovan McGrowder, Sophia Bryan

**Affiliations:** ^1^Department of Basic Medical Sciences, Biochemistry Section, Faculty of Medical Sciences, The University of the West Indies, Mona, Kingston 7, Jamaica; ^2^Department of Pathology, Faculty of Medical Sciences, The University of the West Indies, Mona, Kingston 7, Jamaica

**Keywords:** antimicrobial effect, COVID-19, inhaled nitric oxide, nanoparticles, nitric oxide

## Abstract

Although the World Health Organization has declared that the coronavirus disease (COVID-19) is not a public health emergency of international concern anymore, it has negatively impacted the world, and effective treatment for this pandemic remains a major priority. Vaccine effectiveness has been a matter of concern given the evolution of variants and subvariants of the severe acute respiratory syndrome coronavirus 2 (SARS-CoV-2). Thus, continued protection against SARS-CoV-2 and its variants is still necessary and could work alone or in parallel with vaccinations to treat COVID-19 in the future. Further, findings from in vitro and in vivo studies have noted the effectiveness of high dosages of nitric oxide (NO) as an antimicrobial agent against respiratory pathogens such as bacteria, viruses and fungi. NO has been previously utilized in the management of SARS-CoV and has shown a similar antiviral effect with SARS-CoV-2 in vivo and in vitro. Effective therapy with NO can be used to target several stages of COVID-19 infection to prevent transmission and progression of the disease. The unique properties of NO allow this simple, gaseous molecule to be administered in various forms. NO can be used as an inhalant, in the form of NO donor drugs such as S-nitrosothiols and more recently as NO-releasing nanoparticles (NO-nps). This review summarizes the bioavailability of NO in COVID-19 patients and highlights in vivo and in vitro studies as well as clinical trials with NO administered as a nasal spray, inhalant, or via nanodelivery for therapeutic applications for COVID-19 and other respiratory infections in the future.

## 1. Introduction

COVID-19 quickly spread across the world and thereby achieved the status of a global pandemic. Samples obtained from a patient in Wuhan Jinyintan Hospital on December 30, 2019, tested positive for a novel beta coronavirus [1]. An outbreak followed, and China became the epicentre for a new viral disease that targeted the respiratory system. The virus was recognized as a new coronavirus on January 7, 2020, and was originally named the 2019 novel coronavirus (2019-nCoV) but was later renamed SARS-CoV-2 [2]. SARS-CoV-2 is a member of the coronaviridae family of viruses which also includes SARS-CoV and Middle East respiratory syndrome coronavirus (MERS-CoV) [3]. Coronaviruses (CoVs) contain a nucleocapsid (N) protein with a single-stranded ribonucleic acid (RNA) bound to it and three other structural proteins, namely, the spike (S) protein, the transmembrane (M) protein and the envelope (E) protein [4]. The coronavirus subfamily contains four genera, namely, alpha, beta, delta and gamma CoVs where the alpha and beta CoVs specifically target humans [3]. In comparison to both SARS-CoV and MERS-CoV, SARS-CoV-2 is less pathogenic; however, its infectivity rate is higher [5]. Cells become infected through the attachment of the S protein of SARS-CoV-2 to the angiotensin-converting enzyme 2 (ACE2) receptor [6]. According to the World Health Organization (WHO), up until May 24, 2025, there have been approximately 778 million confirmed cases of COVID-19 recorded internationally and over 7 million deaths [7, 8]. This review is geared toward understanding the antiviral potential of NO against SARS-CoV-2 through assessment of in vivo and in vitro studies as well as highlighting the effect of decreased NO production and bioavailability in COVID-19 patients.

## 2. Physiological Role of Nitric Oxide

Nitric oxide, also called nitrogen monoxide, is a colorless, gaseous molecule with several physiological functions. Nitric oxide was discovered in 1722 by Joseph Priestley and was confirmed years later by Ignarro and colleagues as the endothelial-derived relaxing factor (EDRF) responsible for the regulation of endothelial function in the body [9]. Since then, NO has been widely researched and its significance in the body has been established. Mammalian NO is produced by the enzyme nitric oxide synthase (NOS) of which there are three isoforms: inducible NOS (iNOS), neuronal NOS (nNOS) and endothelial NOS (eNOS) [10]. All three isoforms of the enzyme utilize the substrate L-arginine in the presence of oxygen to produce L-citrulline and NO, with cofactors such as tetrahydrobiopterin (BH_4_) and nicotinamide adenine dinucleotide phosphate (NADPH) [10]. There are differences in the expression and activity of the three different isoforms of the NOS enzyme. The nNOS and eNOS isoforms are continuously expressed in the body; however, this is dependent on calcium ion concentration, whereas iNOS expression is independent of calcium [10]. Nitric oxide is vital for vasodilation and regulates vascular tone and blood flow [11]. It has a significant role in the immune system, in tissue repair and also in neurotransmission in the central and peripheral nervous system [9, 12]. More recently, NO has been recognized for its effect on microorganisms responsible for respiratory diseases. Sorbo et al. reported that administration of NO at a dosage of 160 ppm and above eliminated bacterial colonies of *Staphylococcus aureus* and *Pseudomonas aeruginosa* in vitro and in vivo using an animal model of pneumonia [13]. Additionally, treatment of eight cystic fibrosis patients with 160 ppm of NO resulted in a reduction in the colony-forming units of all bacteria and fungi examined in the sputum [14]. With these findings, future explorations into the application of NO microbial activity are warranted to help in combating the problems of multidrug-resistant pathogens with bacterial pneumonias. Administration of NO with COVID-19 also expands the antiviral action of this gaseous molecule.

## 3. Nitric Oxide Production and Bioavailability in COVID-19

It has been highlighted that mortality due to COVID-19 may be linked to a decrease in NO production and bioavailability [15]. This suggests that an increase in NO availability in the endothelium would have promising effects for COVID-19 patients. In a case–control study, it was discovered that there was a significant reduction in NO metabolites in sixty-eight COVID-19 patients compared to thirty-three healthy normal subjects [16]. The results of the study by Dominic et al. [16] showed a reduction in free nitrite and S-nitrosothiol concentration as well as a decrease in the total nitrite concentration in COVID-19 patients. Montiel et al. [17] conducted an observational study which compared COVID-19 patients in an intensive care unit (ICU) with control subjects and found that there was a decrease in NO bioavailability which was comparable to the progression of the disease. This meant that as the disease transitioned through the mild, moderate and severe stages, the vascular NO concentration decreased accordingly. It was concluded that decreased availability of NO coincident with oxidative stress in the endothelium promoted endothelial dysfunction in the COVID-19 patients [17]. Increased production of reactive oxygen species (ROS) such as superoxide anion and peroxynitrite decreases vessel relaxation and reduces guanylate cyclase expression, resulting in a decrease in the bioavailability and the function of NO [18]. Oxidative stress is an imbalance between oxidants such as ROS and the body's antioxidant defense mechanisms which include enzymatic antioxidants (superoxide dismutase, catalase and glutathione peroxidase) and nonenzymatic antioxidants (vitamins A, C, E and glutathione) [19]. As a result, there is more production of these oxidants which leads to a disruption of redox signaling, deleterious effects on biomolecules and molecular damage which can induce inflammatory immune responses. In normal conditions, redox signaling by ROS compounds is important for cellular functioning and in the immune system [19, 20]. Oxidative stress may be promoted by several viral infections which may lead to an increase in free radical generation and the depletion of important antioxidants [21]. With viral infections, as in the case of COVID-19, there is an overproduction of NO due to the upregulation of iNOS by proinflammatory cytokines such as interferon-γ [22]. Sixty-eight COVID-19 patients with acute respiratory distress syndrome (ARDS) showed a noteworthy decrease in soluble eNOS concentration compared to the twenty-one COVID-19 non-ARDS patients [23]. The reduction in eNOS concentration meant decreased production of NO in the endothelium with increased conditions of oxidative stress. These findings, therefore, provide evidence which supports the postulation that there may be a decrease in endothelial NO production in patients affected with COVID-19.

## 4. L-Arginine Bioavailability in COVID-19

COVID-19 was thought to be a respiratory disease with severe pulmonary consequences in the first instance. However, the disease has been linked to several cardiovascular complications as well due to its impact on endothelial cells [18]. Infection of endothelial cells occurs through binding of SARS-CoV-2 to the ACE2 receptor on the cell membrane [18]. Consequently, viral entry and replication occur in endothelial cells ultimately leading to endothelial dysfunction. Durante [24, 25] reported that upregulation of the arginase enzyme in COVID-19 alters L-citrulline and NO production from the substrate L-arginine and favors the formation of ornithine, polyamines and proline. It was discovered that the expression of the arginase-1 (Arg1) gene was upregulated in COVID-19 through the assessment of twenty-one COVID-19 patients [26]. Upregulation of the Arg1 gene increases the production of the Arg1 enzyme which then increases the utilization of arginine as the substrate. The reduced NO production could be a major cause of the endothelial dysfunction as well as the immune dysfunction observed in patients with COVID-19. In an observational study by Rees et al. [27], a comparison of healthy individuals with COVID-19 patients showed lower bioavailability of L-arginine in the COVID-19 patients. The evidence of upregulation of the Arg1 gene in COVID-19 as well as the reduction in L-arginine concentration provides greater insight into the reduction of NO bioavailability. According to Adebayo et al. [28], there is competition between the arginase enzyme and NOS for the L-arginine substrate; even though the affinity of NOS for L-arginine is higher, arginase has a faster rate of reaction with the substrate. Increased utilization by the arginase enzyme therefore depletes the concentration of the amino acid L-arginine, which in turn decreases NO production and thereby causes a reduction in antiviral activity in the endothelium. In a placebo-controlled clinical trial, it was found that oral administration of 1.66 g of L-arginine twice daily to forty-eight COVID-19 patients reduced the length of stay in the hospital as well as decreased respiratory assistance compared to fifty-three placebo patients [29]. This study therefore showed that supplementation with arginine had a significant therapeutic effect and that the possible mechanism could be through increased NO production. In another clinical study that also supplemented 1.66 g of L-arginine in addition to 500 mg of vitamin C twice daily, it was found that the supplementation improved endothelial function and increased serum L-arginine concentration in adults with long COVID-19 [30].

## 5. In Vivo and In Vitro Studies With Nitric Oxide in the Treatment of COVID-19

Tang et al. conducted in vivo experiments to observe the changes in NO levels using a near-infrared-II fluorescent molecular nanoprobe (OSNP) in mouse models infected with SARS-CoV-2 [31]. A positive association was observed for the level of NO to the progression of the SARS-CoV-2 infection by in vivo visualization of the lung tissues of the mouse model. Further work done by Tang and colleagues involving immuno-histochemical analyses revealed that an improvement in the NO level correlated to an increase in iNOS and not eNOS. The research group postulated that such a finding may possibly be a pathological mechanism by which NO features in COVID-19 [31]. In another study, Michaelsen et al. [32] conducted experiments to determine the safety of prolonged in vivo administration of a high dose (160 ppm) of inhaled NO (iNO) in Yorkshire pigs. From the study, it was reported that continuous high dose of iNO with methylene blue for over 6 h showed no significant changes in lung function or inflammatory markers of pulmonary or systemic injury compared with the control animals [32]. Hence, it was concluded from the study that a high dose of iNO delivery may be clinically feasible and safe. Therefore, further investigations should be done as this type of therapy could potentially be used as a treatment for respiratory infections such as SARS-CoV-2.

An in vitro study which involved infection of African green monkey (Vero E6) cells with the SARS-CoV strain Frankfurt-1 (FFM-1) reported that the NO donor, S-nitroso-N-acetylpenicillamine (SNAP), inhibited viral replication and increased the cell survival rate [33]. The inhibitory concentration of SNAP was approximately 222 μM which facilitated the release of between 30 and 55 μM of NO [33]. In another study, Åkerström et al. [34] confirmed the results of Keyaerts and colleagues with evidence that SNAP dose-dependently inhibited the replication cycle of SARS-CoV within Vero E6 cells and also repressed the production of viral RNA and protein. It was postulated that the NO released from SNAP had a direct antiviral effect as evidenced by a reduction in the viral progeny [34]. Moreover, it was found that induction of iNOS in the Vero E6 cells also inhibited the replication cycle of the virus through NO production, which confirmed that both endogenous and exogenous NO inhibited SARS-CoV [34].

The mechanism by which NO was able to inhibit SARS-CoV replication was discovered a few years later by Åkerström and colleagues. NO inhibited RNA production in the early stage of the SARS-CoV life process [35]. It was also reported that NO decreased palmitoylation of the S protein of SARS-CoV which prevented membrane fusion between the protein and the ACE2 receptor [35]. Lack of membrane fusion between the S protein and the ACE2 receptor thus averted entry of the virus into the host cell and prevented SARS-CoV replication.

**Figure 1:**
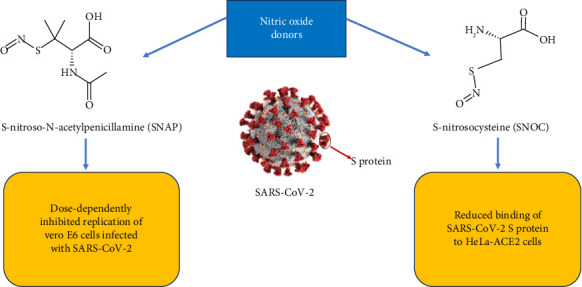
Antiviral effect of nitric oxide donors on SARS-CoV-2 infected cells.

Nitric oxide has also shown potential as an antiviral agent against SARS-CoV-2. Administration of SNAP in an in vitro model with Vero E6 cells was shown to inhibit the replication of SARS-CoV-2 in a dose-dependent manner [36]. This was similar to the effect that SNAP had on SARS-CoV and highlighted the potential of NO treatment against the novel coronavirus SARS-CoV-2 (Figure 1). However, results of a recent in vitro study concluded that short-term and long-term exposure of SARS-CoV-2-infected Vero E6 cells to NO gas had no significant effect on viral replication [37]. Rousseaud et al. [37] therefore concluded that NO gas whether low dose (10 or 40 ppm) or high dose (80 or 160 ppm) had no antiviral effect on SARS-CoV-2 in vitro. This finding was in contrast to the result obtained by Akaberi and colleagues in 2020 [36]. However, it is important to note that the study by Akaberi et al. [36] utilized SNAP as the source of NO, whereas Rousseaud et al. utilized NO gas [37]. Experimentation with NO donors as the source of NO has shown positive outcomes with both SARS-CoV and SARS-CoV-2 in vitro. Therefore, the method of administration of NO is an important factor for in vitro studies with SARS-CoV-2.

It has been stated that the antiviral activity of NO is through S-nitrosylation of the cysteine residues of viral enzymes [22]. This highlighted S-nitrosylation as the mechanism through which NO inhibits viruses. Studies have shown that SARS-CoV-2 inhibition with NO occurred through S-nitrosylation of viral proteases and also S-nitrosylation of the ACE2 receptor of the host [36, 38]. Oh et al. [39] conducted experimentation which included the exposure of HeLa-ACE2 cells to the NO donor, S-nitrosocysteine (SNOC), followed by the addition of the SARS-CoV-2 S protein (Figure 1). The results showed a marked reduction in the binding of the S protein to the HeLa-ACE2 cells and supported the hypothesis that SNOC would bind to the ACE2 receptor to form SNOC-ACE2, thereby preventing attachment of the S protein to the receptor [39]. The second proposed mechanism by which NO inhibits the replication of SARS-CoV-2 is by reducing viral RNA in the early phases of viral replication, and this affects one or both cysteine proteases of SARS-CoV-2 [40]. S-nitrosylation of the ACE2 receptor and specific proteases of SARS-CoV-2 therefore provide a mechanistic understanding of the effects of NO in COVID-19 and highlight a targeted approach to new treatment strategies using specific NO donors.

## 6. NO Nasal Spray (NONS) and the Treatment of COVID-19

The spread of SARS-CoV-2 has declined globally, but the emergence of several strains increases the importance of treating the disease effectively. Treatment for COVID-19 requires a combination of therapeutic approaches to target various stages of infection rather than a single effective treatment [41]. However, NO has the capability to target several stages of SARS-CoV-2 infection leading to a comprehensive treatment strategy.

COVID-19 can be treated by means of a NONS, and there have been a few studies that have investigated the clinical potential of such a therapy. The clinical efficacy of the NONS manufactured by the global pharmaceutical company SaNOtize was investigated in a human randomized, double-blind, placebo-controlled Phase 2 UK clinical trial of 79 confirmed COVID-19 patients. The NONS was effective in considerably decreasing the SARS-CoV-2 levels particularly in those with elevated viral loads [42]. The researchers found a 95% decline in the average viral load log within 24 h and 99% in 72 h. The authors concluded that SaNOtize's NONS signifies a nontoxic and active antiviral therapy that could thwart the spread of COVID-19 and reduce the course and disease severity [42].

**Figure 2:**
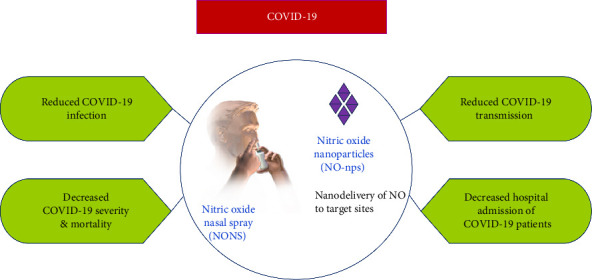
Potential benefits of nitric oxide nasal spray (NONS) and nitric oxide nanoparticles (NO-nps) in COVID-19 patients.

In a clinical trial, Winchester et al. [43] reported that administration of NONS up to six times daily for nine days in forty adult patients with mild COVID-19 lowered SARS-CoV-2 RNA and relieved symptoms compared to a matched placebo group. Additionally, a randomized Phase III clinical trial involving 306 adult Asian patients having mild COVID-19 within the age range of 18–70 years was conducted using NONS [44]. The purpose of the trial was to evaluate the effectiveness of NO to eliminate SARS-CoV-2 RNA from the nasal passages of the trial participants through self-administration of NONS six times daily during a week [44]. Evaluation of the study primarily focused on persons who were at a high risk of COVID-19 disease progression. This high-risk group was defined as individuals who were unvaccinated, older than 45 years of age, or had one or more comorbidities. From the trial, Tandon et al. [44] reported a 93.7% and 99.0% reduction in the concentration of SARS-CoV-2 RNA at 24 and 48 h, respectively. The determination of SARS-CoV-2 RNA in the nasal cavity for mild COVID-19 patients was performed via the reverse transcription polymerase chain reaction (RT-PCR) after a nasal swab was conducted [44] (Table 1). Both studies yielded similar results which highlighted the efficacy of NONS against COVID-19 transmission through the reduction of SARS-CoV-2 RNA in the nasal cavity. From these trials, it was concluded that the use of NONS could possibly decrease COVID-19 infection, hospital admissions, severity and transmission (Figure 2). As such, these findings provide supporting evidence that NONS may potentially reduce the risk of COVID-19 progression in patients [43, 44]. It was stated by Ignarro [48] that nasal administration of NO has a greater possibility of interaction with SARS-CoV-2 in the lungs which allows for inhibition of viral replication or simply the destruction of the virus. Therefore, NONS provides an early intervention strategy in the lowering of SARS-CoV-2 RNA which has beneficial effects in reducing disease transmission.

## 7. iNO and the Treatment of COVID-19

In addition to the utilization of NONS, treatment using iNO has also been tested. iNO was regarded as the chief vasodilator to produce significant selective respiratory vasodilation [49]. Over the years, there have been a number of clinical research studies that reported on the efficacy of iNO in a number of diseases of children and adults [45]. iNO has been used to manage pulmonary hypertension as well as hypoxia in pediatric and adult patients [46] (Table 1). There has been the use of iNO as a therapeutic agent for the treatment of COVID-19 as it induced bronchodilation which resulted in enhanced oxygen delivery to the alveoli while decreasing inflammatory cell-mediated lung damage [50]. Alqahtani and colleagues conducted a systematic review and meta-analysis of 17 studies that examined the use of iNO in the management of COVID-19 patients and reported that the dosage ranged from 9 to 160 ppm over a duration of 30 min to 5 days [47]. iNO was delivered mainly via mechanical ventilators to intubated patients experiencing ARDS. Furthermore, in six of the studies, iNO when administered was combined with angiotensin receptor blockers or ACE inhibitors and other vasodilators such as iloprost, inhaled epoprostenol and almitrine [47] (Table 1). Recently, Di Fenza and colleagues carried out a Phase II multicentred and randomized trial investigating the effect of high-dose iNO (80 ppm) on hypoxemia in 193 patients with COVID-19 acute respiratory failure. There was an improvement in the mean partial pressure of oxygen/fraction of the inspired oxygen (P/F) ratio (PaO_2_/FiO_2_ ratio) of COVID-19 patients undergoing iNO treatment after 2 days compared with the control group receiving usual care [51] (Table 2).

Inhaled nitric oxide treatment in ARDS patients (mild to moderate) with COVID-19 improved the condition without any reported toxicity [59]. The lack of toxicity of iNO to patients increased the benefits associated with the treatment. In this retrospective study, Abman et al. [59] reported an increase in the P/F ratio from 136.7 initially to 151.8 for a total of thirty-four patients after 72 h of iNO treatment. The increased P/F ratio thus indicated improvement in lung function for these patients. iNO activates soluble guanylyl cyclase which in turn increases the production of cyclic 3′–5′-guanosine monophosphate (cGMP) in smooth muscle cells of the lungs and thus causes vasodilatory effects [59].

There are studies that investigate the clinical utility of iNO in the management of severe COVID-19 patients with refractory hypoxemia [5]. In a recent study, iNO at a concentration of 15–20 ppm was administered to 10 patients with severe COVID-19 pneumonia having refractory hypoxemia in a tertiary respiratory ICU. iNO therapy caused improvements in arterial oxygen saturation, PaO_2_, P/F ratio and shunt fraction. The authors noted that the improvement in these indices did not translate into decreased mortality possibly due to the severity of COVID-19 pneumonia and the limited duration of iNO therapy [55] (Table 2). In an earlier reported multicentre, retrospective cohort study, the researchers investigated the use of iNO therapy in the management of 815 critically ill adult COVID-19 patients having moderate-to-severe ARDS. There were improved oxygenation parameters such as P/F ratio, FiO_2_ requirements, oxygenation index and PaO_2_ within 24 h of iNO administration. Similar to other studies, the authors found no mortality benefits and that iNO therapy was associated with a higher risk of acute kidney injury, pneumonia and longer ICU length of stay [54] (Table 2). A multicentre retrospective cohort study of 300 COVID-19 patients with ARDS of varying severity (2% mild, 37% moderate and 61% severe) showed similar findings of improved oxygenated parameters particularly in the most severe cases post iNO administration. Unlike other studies, the benefits of iNO therapy were associated with better survival [52] (Table 2). Other studies have reported similar findings of improved oxygenated parameters although the effect of iNO therapy on survival was low [53, 56].

Pregnant women experiencing COVID-19 may develop pneumonia which is life-threatening as it may rapidly progress to hypoxic respiratory failure necessitating cardiopulmonary support and hospital admission [60]. There is also an increasing risk of admission to ICU, requirement of extracorporeal membrane oxygenation, or mechanical ventilation as well as obstetric difficulties such as preterm delivery, preeclampsia and stillbirth [61]. A positive effect of iNO was also seen in pregnant women with COVID-19. Valsecchi et al. [57] found that NO (up to 200 ppm) administered twice daily to twenty pregnant women with severe COVID-19 pneumonia decreased the length of hospitalization as well as the need for supplemental oxygen. The use of iNO in pregnant women with COVID-19 given the lack of adverse effects proves advantageous to both the mother and the child. A study in a Massachusetts General Hospital documented that administration of high-dose nitric oxide (160–200 ppm) twice daily in six pregnant women with severe COVID-19 enhanced respiratory rate and oxygenation [58]. The results of this study therefore correspond with those of Valsecchi et al. and further promote the use of iNO also in pregnant women with the severe form of the disease without adverse effects. It was also reported that after 28 days of hospitalization, five of the pregnant women received negative RT-PCR tests for SARS-CoV-2 [58] (Table 2). iNO therapy is related to improved oxygenation and respiratory rate for pregnant patients with severe or life-threatening COVID-19 and points to its potential benefits in providing better outcomes with future investigations involving prospective randomized trials [62]. Overall, iNO administration allows for the treatment of various stages of COVID-19 infection and also facilitates safe treatment for pregnant patients.

iNO has been reported to relax pulmonary vessels and to increase pulmonary blood oxygenation. This has been the only type of NO-based therapy that has been approved by the US Food and Drug Administration (FDA) for treating newborns with severe and persistent pulmonary hypertension. Investigations into its use for adults have been ongoing. However, a limitation of using iNO has been that an optimal threshold has not yet been determined as it is believed that there may be long-term implications such as neurodevelopmental problems. Several methods including computational modelling have been developed to try to quantify the amounts of NO being delivered as this may correlate to its therapeutic effects and would be important for deciding on the dosages for clinical applications [63]. Due to the shortage of ventilators, FDA approval was granted for emergency access to use iNO to treat mild to moderate COVID-19 patients. Phase II trials (NCT04305457, NCT04306393 and NCT04312243) are ongoing to assess the use of NO for treatment or prevention of COVID-19 [40]. New NO delivery devices and technology systems are under development to provide bedside solutions for iNO therapy. Such offerings provide stable, portable and on-demand generation of NO, thereby replacing traditional cylinder-based systems which are associated with high financial costs and logistic challenges [50].

Additional safety issues that may arise from treatment with iNO is the formation of methaemoglobin and nitrogen dioxide (NO_2_). A well-known potential complication of iNO treatment in patients is methaemoglobinemia which results from either an increase in methaemoglobin or a decrease in its breakdown. When NO oxidizes heme iron in the ferrous (Fe^2+^) to the ferric (Fe^3+^) state, this results in methaemoglobin being formed. Methaemeglobin has a high affinity for oxygen; however, it has a decreased oxygen-carrying capacity due to its inability to bind oxygen. This results in a decrease in the unloading of oxygen, thereby reducing oxygen delivery to tissues in the body [64]. Methaemoglobinemia may occur during iNO administration due to several reasons which may include errors when monitoring NO levels, in the delivery of NO, unintentional overdosing and the absence of the enzyme methaemoglobin reductase which is responsible for converting methaemoglobin back to haemoglobin [65]. It has been reported that the normal physiological level of methaemoglobin in the blood is less than 2% [66]. However, methaemoglobin levels above 2% can lead to methaemoglobinemia with levels greater than 70% causing death [66].

Furthermore, inhaled nitric oxide has the ability to react with oxygen to produce nitrogen dioxide (NO_2_) which is very toxic to the body. With the use of the compressed gas delivery system, NO can be mixed with oxygen resulting in the codelivery of NO_2_ [67]. Changes in pulmonary function are evident when healthy subjects are exposed to 2–3 ppm NO_2_ and can occur at far lower concentrations in asthmatic subjects. More severe NO_2_ exposures (> 25 ppm) can be fatal and cause conditions such as pneumonitis, bronchiolitis obliterans and pulmonary edema [68]. It is therefore important to minimize NO_2_ levels with inhaled nitric oxide therapy. According to the Occupational Safety and Health Administration, the permissible exposure limit for NO_2_ is 5.0 ppm (9 mg/m^3^), whereas the recommended exposure limit is 1.0 ppm (1.8 mg/m^3^) [69]. The National Institute for Occupational Safety and Health also recommended an exposure limit of 1.0 ppm for NO_2_ [70].

In the study by Safaee et al., the maximum level of methaemoglobin measured for iNO at 160 ppm twice daily for 30 min was 4.7% and the maximum NO_2_ measured was 1.5 ppm [58]. There were no reports of termination of treatment due to high methaemoglobin or NO_2_ levels. Gianni et al. also reported on the use of 160 ppm twice daily for 15 min with NO_2_ levels ranging from 0.70 to 0.75 ppm for pressurized NO delivery and 0.74–0.88 ppm for delivery via electric NO generators [71]. Methaemoglobin levels reached a maximum of 1.98% for pressurized NO delivery and 1.89% for electric NO delivery amongst these participants. Furthermore, Wiegand et al. found that NO_2_ was below 2 ppm and the maximum methaemoglobin level was 2.0% (1.7%–2.3%) for treatment with 160 ppm iNO twice daily for 30 min [72]. The evidence suggested that iNO treatment for 15 or 30 min at a high dose of 160 ppm showed positive safety outcomes of methaemoglobin and NO_2_ concentration.

## 8. Nitric Oxide–Releasing Nanoparticles and COVID-19

There are challenges with delivering NO due to its unregulated presence throughout the blood circulation and short half-life of just a few seconds. With the advancement in biotechnology, NO donors can be physically embedded or chemically conjugated with nanocarriers to deliver NO [40]. Incorporating nitric oxide with nanoparticles expands its application in the medical field and provides potential benefits such as increased stability as well as precise and controlled systemic release. This method of drug delivery which utilizes nanoparticles, commonly called nano-delivery, has become of great significance to medicine and even more so to NO research. Nanoparticles are ultrasmall entities that generally range from 1 to 100 nm in size [73]. Several categories of nanoparticles exist such as liposomes, dendrimers, as well as those that are metal-, lipid- and polymer-based [74]. These nanoparticles can be allied to specific drugs to facilitate targeted delivery in medical applications. Schairer et al. [75] outlined the use of NO-releasing nanoparticles (NO-nps) which generate NO from nitrite under thermal conditions. Additionally, Pieretti et al. reported the use of NO donors (organic nitrates, S-nitrosothiols, metal complexes and N-diazeniumdiolates) combined with nanomaterials in the form of copper, silver and polymeric nanoparticles and the beneficial antibacterial activity obtained from such molecules [76].

In a study by Williams et al. [77], it was reported that intra-arterial administration of 10 mg/kg of NO-nps to mice with lipopolysaccharide-induced endotoxemia improved acute inflammatory condition and the survival rate of mice [77]. Importantly, there was a reduction in proinflammatory cytokines such as interleukin 1 (IL-1), interleukin 6 (IL-6), interleukin 12 (IL-12) and tumor necrosis factor alpha (TNF-α) in the serum of treated mice compared to the control [77]. The results of the study highlighted the potential of treatment with NO-nps to prevent the cytokine storm that accompanies COVID-19 [77]. Shurbaji et al. [78] reported that the use of three different formations of NO-releasing hydrogel nanocomposites-based nanoparticles (NO-RPs) in an in vitro system with lung epithelial cells from rats improved cell viability. This in vitro system was incredibly significant as it modelled damage from mechanical ventilation during the treatment of ARDS. Findings from the study showed that NO-releasing nanoparticle 1 (NO-RPs1) which delivered the lowest concentration of NO of the three nanoparticles had the greatest protective effect on the lung epithelial cells [78]. This study highlighted that treatment with NO nanoparticles which released a low concentration of NO could be useful in the treatment of ARDS resulting from COVID-19.

A review of the literature has provided no evidence of the use of NO nanoparticles as yet in clinical trials with COVID-19 patients. However, other nanoparticles have been utilized in clinical trials such as silver nanoparticles (AgNPs). A randomized clinical trial was recently conducted with AgNPs in COVID-19 pneumonia patients in Kolkata, India [79]. According to Wieler and colleagues [64], intravenous administration of AgNPs at a total dosage of 5.4 mg/day per patient decreased the need and duration for supplementation with oxygen as well as decreased patient mortality. Another clinical trial conducted for 9 weeks in the General Tijuana Hospital, Mexico, found that utilization of a mouthwash and nose rinse solution with AgNPs significantly reduced the infection rate of healthcare workers interacting with COVID-19 patients [80]. Although the mechanism for reducing infectivity was unknown, it was proposed that inhibition of the attachment of SARS-CoV-2 viral proteins to host cell receptors as well as to viral genetic material could be a possible mechanism of action [80]. This proposed mechanism of action would be similar to what has been evidenced for in vitro studies with SNAP and SNOC [36, 39]. Studies have been completed with the use of metal nanoparticles which are worthy of note and the combination of such nanoparticles with NO could be beneficial in targeting the SARS-CoV-2 virus.

With the application of nanotechnology techniques, nanocarriers can be designed in such a way that therapeutic amounts of NO are delivered in a sustained and controlled manner to target sites, for example, at the site of the viral infection and even more at specific concentrations (Figure 2). Another important feature of using nanotechnology is that the stability of NO is further increased when NO donors are combined with nanoparticles. This is vital due to the numerous roles that NO plays in the body and being a free radical with a short half-life of less than 5 seconds [76]. Notwithstanding, there are challenges in using NO nanoparticles in which they all face the same issues. The foremost being that of safety concerns for its clinical application to treat COVID-19 patients. The process of producing nanocarriers is a rather intricate one which involves the use of numerous chemicals that are potentially hazardous and may not have been given approval to be used in humans. Problems of toxicity may arise even with FDA-approved liposomes when they are used in combination with different clinical drugs. Nanoparticles may result in other toxicity problems as they may impact biodistribution and interactions with cells and biomolecules due to their physicochemical properties such as size, shape and surface charge. Also, the concentration of NO may lead to toxicity issues as NO is a highly reactive molecule that can destroy healthy tissues and cells [40]. It has been suggested by Rana that high doses of these nanoparticulates have the potential to cause adverse side effects to humans as a result of an off-targeting feature that could possibly be more severe than an infection by SARS-CoV-2 [81]. Furthermore, the formation of a protein corona is possible due to the interaction of nanocarriers once in the blood circulation [81]. Therefore, minimum effective dosages of NO should be determined to treat COVID-19 so that potential toxicity problems do not become an issue. The optimal amount of NO required may differ with the phase and severity of COVID-19, and so more research will need to be done in these areas. Hence, further studies involving the therapeutic mechanisms of NO will pave the way in giving theoretical guidance for the clinical application of NO nanoparticles. The utilization of NO nanoparticles has the potential of someday becoming part of a lifesaving therapy against possible COVID-19 reoccurrence and for any other viral infections [40].

To date, the effects of using NO nanoparticles in treating patients with COVID-19 are not yet known, as such a cautious approach should be taken in the clinical application of these drugs. It is important that clear guidelines for their development and use be established which should be guided by rigorous research. This is especially vital as it relates to safety and toxicity concerns in using nanoparticles and even more so NO nanoparticles for COVID-19 treatment. Research studies should determine the most suitable mode of delivery as well as the optimal dosage required to treat the different stages of illness severity from mild to moderate and severe cases of COVID-19 while still being effective but safe for human use. Other considerations should be to investigate any potential drug interactions between these NO nanoparticles and drugs that may affect the immune system and other antiviral drugs. Preclinical and clinical studies are needed therefore to establish the role of NO nanoparticles in COVID-19 treatment [40].

## 9. Conclusion

Nitric oxide is a promising solution to the COVID-19 problem. The beneficial antiviral activity of NO has already been evidenced with SARS-CoV with similar results being observed for SARS-CoV-2 in vivo and in vitro. A reduction in NO bioavailability has been reported in COVID-19 patients in which there was an upregulation of the Arg1 enzyme, resulting in the depletion of L-arginine and decreased eNOS activity. Studies have shown though that there are beneficial effects of a NONS in the lowering of SARS-CoV-2 RNA, as well as an improvement in oxygenation and the respiratory rate with iNO treatment. Inhaled nitric oxide also resulted in improved respiratory outcomes for several categories of COVID-19 patients (mild, moderate and critically ill) and was even beneficial to pregnant patients. The use of nitric oxide nanoparticles further expands its application against viral diseases. The possible use of NO-nps for future treatment of COVID-19 may allow for the controlled delivery of low-dose NO to targeted areas. Other nanoparticles have been used in clinical trials which have proven effective without adverse effects to patients. However, there needs to be consideration for toxicity as well as effective dosage of nitric oxide nanoparticles given the lack of use in a clinical setting. Further in vivo studies and clinical trials should be encouraged specifically with these nanoparticles to increase our knowledge of COVID-19 and to transform treatment for possible reoccurrences of this disease in the future. The activity of NO against bacterial, fungal and viral respiratory pathogens has been noteworthy. Therefore, the antimicrobial action of NO could play a vital role in the defense against future outbreaks of COVID-19 and other respiratory diseases.

## Figures and Tables

**Table 1:** Nitric oxide nasal spray, inhalation therapy and clinical outcomes of COVID-19 patients.

Study design and number of participants	Drug(s) administered and duration	Main study findings	Remarks/conclusion	References
Clinical trial of 80 adults (18–70 years) who were isolated with mild COVID-19 infection confirmed by a laboratory test	Participants were randomized 1:1 to receive NONS (*n* = 40) placebo (*n* = 40). The nasal sprays were self-administered 5-6 times daily (two sprays per nostril/dose, 120–140 μL of solution/spray) for 9 days	Patients in the NONS treatment arm demonstrated viral loads that were lower at Days 2 and 4 than those on placebo, and symptom resolution was also found to be faster on NONS treatment than on placebo	Treatment with NONS was found to be effective and safe in reducing the viral load in patients with mild, symptomatic COVID-19 infection	[43]
A randomized, double-blind, multicentre, parallel group, placebo-controlled Phase III clinical trial of 30 adults (18–70 years) with mild symptomatic COVID-19 [randomization was 1:1, NONS (*N* = 153) versus placebo (*N* = 153)]	NO generated by a nasal spray (NONS) was self-administered six times daily as two sprays per nostril (0·45 mL of solution/dose) for 7 days	Secondary endpoint assessments showed a greater proportion of patients receiving NONS (82·8%) cleared SARS-CoV-2 (RT-PCR negative) by end of treatment compared to placebo (66·7%, *p*=0.046)	Use of NONS in patients recently infected with SARS-CoV-2 accelerates nasal virus clearance	[44]
Prospective study of 42 patients (26–77 years) with pulmonary hypertension from primary and secondary causes	Patients received inhaled nitric oxide (iNO) and their response to iNO was defined by a decrease of > or = 20% in mean pulmonary artery (PA) pressure or pulmonary vascular resistance (PVR)	Mean PVR and PA pressures were lesser during NO inhalation in all patients with pulmonary hypertension	NO is a safe and effective screening agent for patients with primary or secondary pulmonary hypertension	[45]
Prospective study of 6 infants with persistent pulmonary hypertension of the newborn	Infants received 80 ppm of NO with fraction of inspired oxygen (F_1_O_2_) of 0.9 for 30 min	Inhaled NO treatment rapidly and considerably increased preductal oxygen saturation (SpO_2_) and in five subjects postductal SpO_2_ and oxygen tensions were also increased	Low levels of inhaled NO have a significant role in reversing hypoxemia due to pulmonary hypertension of the newborn	[46]
Systematic review of 17 studies (including 712 COVID-19 patients) of which 8 undergo meta-analysis	The review investigated whether iNO has significant benefit compared with standard treatment (without iNO) in the management of COVID-19-ARDS. The dosage of iNO in patients with COVID-19 ranged between 9 and 160 ppm over a duration between 30 min and 5 days	Eight (47%) of the included studies reported response to iNO in COVID-19 patients suffering from ARDS. Response was described as improvement in oxygenation as measured by the P/F ratio, PaO_2_ and SpO_2_, among others, following iNO administration	Inhaled NO therapy is valuable in the treatment of hypoxemia in COVID-19 patients and may improve systemic oxygenation in patients with COVID-19-ARDS	[47]

**Table 2:** Inhaled nitric oxide and respiratory outcomes of COVID-19 patients.

Study design and number of participants	Drug(s) administered and duration	Safety outcomes: methaemoglobin (MetHb) and nitrogen dioxide (NO_2_) levels	Main study findings	Remarks/conclusion	References
Multicentre retrospective cohort study of 300 patients COVID-19-related acute respiratory distress syndrome (C-ARDS)	Inhaled NO at different time points (within 6 h, within 24 h) with a median duration of 2.8 days and a median dosage of 10 ppm	Unreported	PaO_2_/FiO_2_ ratio improved (by 20% or more) in 45.7% of patients at 6 h after initiation. Patients' ECMO criteria (severe cases) showed improvements (32/52, 51.6%) with better survival	iNO enhanced arterial oxygenation in patients with C-ARDS particularly in severe cases which was associated with improved survival	[52]
A phase II, multicentre, single-blind, randomized, controlled, parallel-arm trial comprised 193 mechanically ventilated adults with COVID-19 pneumonia	Participants in the treatment arm received inhaled NO at 80 ppm for the first 48 h after enrollment	The highest level of MetHb at 80 ppm was 2.2% (1.5–3.0). The NO_2_ highest level at 80 ppm was 1.0% (1.0%–1.8%)	Larger proportion of participants (27.7%) in the treatment group attained a PaO_2_/FiO_2_ > 300 mmHg than controls (17.2%) with no serious adverse events	High-dose iNO caused an improvement of PaO_2_/FiO_2_ after 2 days, and there was no improvement in survival compared with usual care	[51]
Prospective cohort study of 10 patients with severe COVID-19 pneumonia (and refractory hypoxaemia) in a tertiary respiratory intensive care unit	iNO mixture introduced into the inspiratory limb of the ventilator tubing at a concentration of 15–20 ppm	Unreported	Mean increase in PaO_2_/FiO_2_ and decrease in shunt fraction (20%) but improvements did not result with better survival	Improved respiratory parameters but no mortality benefit to patients	[53]
Multicentre, retrospective cohort study of 815 critically ill adult patients with confirmed COVID-19	An initial dose of iNO (20 ppm) was applied to patients	Unreported	Significantly improved oxygenation parameters such as PaO_2_, FiO_2_ requirement, P/F ratio and oxygenation index 24 h after iNO administration	Improved oxygen parameters in critically ill COVID-19 patients with moderate-to-severe ARDS but no survival benefit	[54]
Retrospective study of 16 patients with ARDS due to COVID-19 admitted to the intensive care unit	An iNO dose of 20 ppm administered with a mean duration of treatment of 3.5 days	Unreported	Improved oxygen parameters with a significant increase in PaO_2_/FiO_2_ values	Better oxygenation in patients with severe ARDS, but no mortality benefit	[55]
A retrospective study of 35 COVID-19 patients admitted to ICU with at least moderate ARDS	Patients were treated with 20 ppm iNO for an average of 146.4 (80.8) h, with the exception of one patient treated with 40 ppm	Unreported	Improved oxygenation (PaO_2_/FiO_2_ ratio and OI) reduces dead space ventilation post iNOS administration	iNO may be helpful in patients with COVID-19 with refractory hypoxemia	[56]
Retrospective cohort study of 71 pregnant women hospitalized with severe COVID-19 pneumonia	iNO200 was delivered twice per day for at least 30 min per administration	Mean MetHb was 2.8% (0.4%–5.3%), and NO_2_ was 1.8 ppm (1.14–2.5 ppm)	Patients in the iNO200 cohort were observed to have significantly shorter ICU and hospital length of stay as well as days free from oxygen supplementation (*p* < 0.001)	Pregnant COVID-19 patients admitted to ICU with severe pneumonia had reduced oxygen supplementation needs and shorter stay in hospital	[57]
Interventional study of 29 nonintubated COVID-19 patients	iNO at 160 ppm administered for 30 min twice daily	MetHb peaked at 2.5% (2.0%–3.0%). NO_2_ was unreported	Improved oxygenation and respiratory rate with no observed adverse events such as hypotension and hypoxemia	Improvement in oxygen parameters with no adverse events	[58]

## Data Availability

The data supporting this narrative review are from previously reported studies, which have been cited within the article.

## References

[1] Gomes C. (2020). Report of the WHO-China Joint Mission on Coronavirus Disease 2019 (COVID-19). *Brazilian Journal of Implantology and Health Sciences*.

[2] Merad M., Blish C. A., Sallusto F., Iwasaki A. (2022). The Immunology and Immunopathology of COVID-19. *Science*.

[3] Rabaan A. A., Al-Ahmed S. H., Haque S. (2020). SARS-CoV-2, SARS-CoV, and MERS-COV: A Comparative Overview. *Infezioni in Medicina, Le*.

[4] Hasöksüz M., Kiliç S., Saraç F. (2020). Coronaviruses and SARS-COV-2. *Turkish Journal of Medical Sciences*.

[5] Rajendran R., Chathambath A., Al-Sehemi A. G. (2022). Critical Role of Nitric Oxide in Impeding COVID-19 Transmission and Prevention: a Promising Possibility. *Environmental Science and Pollution Research*.

[6] Ludwig S., Zarbock A. (2020). Coronaviruses and SARS-CoV-2: A Brief Overview. *Anesthesia & Analgesia*.

[7] World Health Organization (2025). *Covid-19 Cases*.

[8] World Health Organization (2025). *Covid-19 Deaths*.

[9] Ignarro L. J., Buga G. M., Wood K. S., Byrns R. E., Chaudhuri G. (1987). Endothelium-Derived Relaxing Factor Produced and Released from Artery and Vein is Nitric Oxide. *Proceedings of the National Academy of Sciences*.

[10] Luiking Y. C., Engelen M. P., Deutz N. E. (2010). Regulation of Nitric Oxide Production in Health and Disease. *Current Opinion in Clinical Nutrition and Metabolic Care*.

[11] Chen K., Pittman R. N., Popel A. S. (2008). Nitric Oxide in the Vasculature: where Does it Come From and Where Does it Go? A Quantitative Perspective. *Antioxidants and Redox Signaling*.

[12] Antosova M., Plevkova J., Strapkova A., Buday T. (2012). Nitric Oxide—Important Messenger in Human Body. *Open Journal of Molecular and Integrative Physiology*.

[13] Sorbo L. D., Michaelsen V. S., Ali A., Wang A., Ribeiro R. V. P., Cypel M. (2022). High Doses of Inhaled Nitric Oxide as an Innovative Antimicrobial Strategy for Lung Infections. *Biomedicines*.

[14] Deppisch C., Herrmann G., Graepler-Mainka U. (2016). Gaseous Nitric Oxide to Treat Antibiotic Resistant Bacterial and Fungal Lung Infections in Patients With Cystic Fibrosis: A Phase I Clinical Study. *Infection*.

[15] Ozdemir B., Yazici A. (2020). Could the Decrease in the Endothelial Nitric Oxide (NO) Production and NO Bioavailability be the Crucial Cause of COVID-19 Related Deaths?. *Medical Hypotheses*.

[16] Dominic P., Ahmad J., Bhandari R. (2021). Decreased Availability of Nitric Oxide and Hydrogen Sulfide is a Hallmark of COVID-19. *Redox Biology*.

[17] Montiel V., Lobysheva I., Gérard L. (2022). Oxidative Stress-Induced Endothelial Dysfunction and Decreased Vascular Nitric Oxide in COVID-19 Patients. *EBioMedicine*.

[18] Six I., Guillaume N., Jacob V. (2022). The Endothelium and COVID-19: An Increasingly Clear Link Brief Title: Endotheliopathy in COVID-19. *International Journal of Molecular Sciences*.

[19] Birben E., Sahiner U. M., Sackesen C., Erzurum S., Kalayci O. (2012). Oxidative Stress and Antioxidant Defense. *World Allergy Organization Journal*.

[20] Meulmeester F. L., Luo J., Martens L. G., Mills K., van Heemst D., Noordam R. (2022). Antioxidant Supplementation in Oxidative Stress-Related Diseases: What Have we Learned From Studies on alpha-tocopherol?. *Antioxidants*.

[21] Ntyonga-Pono M. P. (2020). COVID-19 Infection and Oxidative Stress: an Under-Explored Approach for Prevention and Treatment?. *The Pan African medical journal*.

[22] Yamasaki H., Imai H., Tanaka A., Otaki J. M. (2023). Pleiotropic Functions of Nitric Oxide Produced by Ascorbate for the Prevention and Mitigation of COVID-19: A Revaluation of Pauling’s Vitamin C Therapy. *Microorganisms*.

[23] Vassiliou A. G., Zacharis A., Keskinidou C. (2021). Soluble Angiotensin Converting Enzyme 2 (ACE2) is Upregulated and Soluble Endothelial Nitric Oxide Synthase (eNOS) is Downregulated in COVID-19-induced Acute Respiratory Distress Syndrome (ARDS). *Pharmaceuticals*.

[24] Durante W. (2022). Targeting Arginine in Covid-19-Induced Immunopathology and Vasculopathy. *Metabolites*.

[25] Durante W. (2023). Glutamine Deficiency Promotes Immune and Endothelial Cell Dysfunction in COVID-19. *International Journal of Molecular Sciences*.

[26] Derakhshani A., Hemmat N., Asadzadeh Z. (2021). Arginase 1 (*Arg1*) as an Up-Regulated Gene in COVID-19 Patients: a Promising Marker in COVID-19 Immunopathy. *Journal of Clinical Medicine*.

[27] Rees C. A., Rostad C. A., Mantus G. (2021). Altered Amino Acid Profile in Patients With SARS-CoV-2 Infection. *Proceedings of the National Academy of Sciences*.

[28] Adebayo A., Varzideh F., Wilson S. (2021). L-arginine and COVID-19: An Update. *Nutrients*.

[29] Fiorentino G., Coppola A., Izzo R. (2021). Effects of Adding L-arginine Orally to Standard Therapy in Patients with COVID-19: a Randomized, Double-Blind, Placebo-Controlled, Parallel-Group Trial. Results of the First Interim Analysis. *eClinicalMedicine*.

[30] Tosato M., Calvani R., Picca A. (2022). Effects of L-arginine plus Vitamin C Supplementation on Physical Performance, Endothelial Function, and Persistent Fatigue in Adults With Long COVID: A Single-Blind Randomized Controlled Trial. *Nutrients*.

[31] Tang Y., Li Y., Wang Z., Huang W., Fan Q., Liu B. (2023). In Situ Noninvasive Observation of Nitric Oxide Fluctuation in SARS-CoV-2 Infection in Vivo by Organic Near-Infrared-II Fluorescent Molecular Nanoprobes. *ACS Nano*.

[32] Michaelsen V. S., Ribeiro R. V. P., Brambate E. (2021). A Novel Pre-Clinical Strategy to Deliver Antimicrobial Doses of Inhaled Nitric Oxide. *PLoS One*.

[33] Keyaerts E., Vijgen L., Chen L., Maes P., Hedenstierna G., Van Ranst M. (2004). Inhibition of SARS-Coronavirus Infection in Vitro by S-nitroso-N-Acetylpenicillamine, a Nitric Oxide Donor Compound. *International Journal of Infectious Diseases*.

[34] Åkerström S., Mousavi-Jazi M., Klingström J., Leijon M., Lundkvist A., Mirazimi A. (2005). Nitric Oxide Inhibits the Replication Cycle of Severe Acute Respiratory Syndrome Coronavirus. *Journal of Virology*.

[35] Åkerström S., Gunalan V., Keng C. T., Tan Y. J., Mirazimi A. (2009). Dual Effect of Nitric Oxide on SARS-CoV Replication: Viral RNA Production and Palmitoylation of the S Protein are Affected. *Virology*.

[36] Akaberi D., Krambrich J., Ling J. (2020). Mitigation of the Replication of SARS-CoV-2 by Nitric Oxide in Vitro. *Redox Biology*.

[37] Rousseaud A., Prot M., Loriere E. S., Katz I., Ramirez-Gil J. F., Farjot G. (2023). Gaseous Nitric Oxide Failed to Inhibit the Replication Cycle of SARS-CoV-2 in Vitro. *Nitric Oxide*.

[38] Sodano F., Cavanagh R. J., Pearce A. K. (2020). Enhancing Doxorubicin Anticancer Activity With a Novel Polymeric Platform Photoreleasing Nitric Oxide. *Biomaterials Science*.

[39] Oh C. K., Nakamura T., Beutler N. (2023). Targeted Protein S-Nitrosylation of ACE2 Inhibits SARS-CoV-2 Infection. *Nature Chemical Biology*.

[40] Wang Z., Jin A., Yang Z., Huang W. (2023). Advanced Nitric Oxide Generating Nanomedicine for Therapeutic Applications. *ACS Nano*.

[41] Dong Y., Shamsuddin A., Campbell H., Theodoratou E. (2021). Current COVID-19 Treatments: Rapid Review of the Literature. *Journal of Global Health*.

[42] Mitchell J. P., Berlinski A., Canisius S. (2020). Urgent Appeal From International Society for Aerosols in Medicine (ISAM) During COVID-19: Clinical Decision Makers and Governmental Agencies Should Consider the Inhaled Route of Administration: A Statement From the ISAM Regulatory and Standardization Issues Networking Group. *Journal of Aerosol Medicine and Pulmonary Drug Delivery*.

[43] Winchester S., John S., Jabbar K., John I. (2021). Clinical Efficacy of Nitric Oxide Nasal Spray (NONS) for the Treatment of Mild COVID-19 Infection. *Journal of Infection*.

[44] Tandon M., Wu W., Moore K. (2022). SARS-CoV-2 Accelerated Clearance Using a Novel Nitric Oxide Nasal Spray (NONS) Treatment: A Randomized Trial. *The Lancet Regional Health-Southeast Asia*.

[45] Krasuski R. A., Warner J. J., Wang A., Harrison J. K., Tapson V. F., Bashore T. M. (2000). Inhaled Nitric Oxide Selectively Dilates Pulmonary Vasculature in Adult Patients With Pulmonary Hypertension, Irrespective of Etiology. *Journal of the American College of Cardiology*.

[46] Roberts J. D., Polaner D. M., Lang P., Zapol W. M. (1992). Inhaled Nitric Oxide in Persistent Pulmonary Hypertension of the Newborn. *Lancet (London, England)*.

[47] Alqahtani J. S., Aldhahir A. M., Al Ghamdi S. S. (2022). Inhaled Nitric Oxide for Clinical Management of COVID-19: A Systematic Review and Meta-Analysis. *International Journal of Environmental Research and Public Health*.

[48] Ignarro L. J. (2020). Inhaled NO and COVID-19. *British Journal of Pharmacology*.

[49] Yu B., Ichinose F., Bloch D. B., Zapol W. M. (2019). Inhaled Nitric Oxide. *British Journal of Pharmacology*.

[50] Kamenshchikov N. O., Berra L., Carroll R. W. (2022). Therapeutic Effects of Inhaled Nitric Oxide Therapy in COVID-19 Patients. *Biomedicines*.

[51] Di Fenza R., Shetty N. S., Gianni S. (2023). High-Dose Inhaled Nitric Oxide in Acute Hypoxemic Respiratory Failure Due to COVID-19: A Multicenter Phase II Trial. *American Journal of Respiratory and Critical Care Medicine*.

[52] Mekontso Dessap A., Papazian L., Schaller M. (2023). Inhaled Nitric Oxide in Patients With Acute Respiratory Distress Syndrome Caused by COVID-19: Treatment Modalities, Clinical Response, and Outcomes. *Annals of Intensive Care*.

[53] Bicakcioglu M., Kalkan S., Duzenci D., Yalcinsoy M., Dogan Z., Ozer A. B. (2023). Inhaled Nitric Oxide as Rescue Therapy in Severe ARDS Cases due to COVID-19 Pneumonia: a Single Center Experience. *European Review for Medical and Pharmacological Sciences*.

[54] Al Sulaiman K., Korayem G. B., Altebainawi A. F. (2022). Evaluation of Inhaled Nitric Oxide (Ino) Treatment for Moderate-to-Severe ARDS in Critically Ill Patients With COVID-19: a Multicenter Cohort Study. *Critical Care*.

[55] van Zyl A. G. P., Allwood B. W., Koegelenberg C. F. N., Lalla U., Retief F. (2023). The Effect of Inhaled Nitric Oxide on Shunt Fraction in Mechanically Ventilated Patients With COVID-19 Pneumonia. *African Journal of Thoracic and Critical Care Medicine*.

[56] Garfield B., McFadyen C., Briar C. (2021). Potential for Personalised Application of Inhaled Nitric Oxide in COVID-19 Pneumonia. *British Journal of Anaesthesia*.

[57] Valsecchi C., Winterton D., Safaee Fakhr B. (2022). High-Dose Inhaled Nitric Oxide for the Treatment of Spontaneously Breathing Pregnant Patients with Severe Coronavirus Disease 2019 (COVID-19) Pneumonia. *Obstetrics & Gynecology*.

[58] Safaee Fakhr B., Wiegand S. B., Pinciroli R. (2020). High Concentrations of Nitric Oxide Inhalation Therapy in Pregnant Patients With Severe Coronavirus Disease 2019 (COVID-19). *Obstetrics & Gynecology*.

[59] Abman S. H., Fox N. R., Malik M. I. (2022). Real-World Use of Inhaled Nitric Oxide Therapy in Patients With COVID-19 and Mild-to-Moderate Acute Respiratory Distress Syndrome. *Drugs in Context*.

[60] Zambrano L. D., Ellington S., Strid P. (2020). Update: Characteristics of Symptomatic Women of Reproductive Age with laboratory-confirmed SARS-CoV-2 Infection by Pregnancy Status-United States, January 22-October 3, 2020. *MMWR. Morbidity and Mortality Weekly Report*.

[61] Kasehagen L., Byers P., Taylor K. (2021). COVID-19-associated Deaths After SARS-CoV-2 Infection During Pregnancy-Mississippi, March 1, 2020-October 6, 2021. *MMWR. Morbidity and Mortality Weekly Report*.

[62] Alvarez R. A., Berra L., Gladwin M. T. (2020). Home Nitric Oxide Therapy for COVID-19. *American Journal of Respiratory and Critical Care Medicine*.

[63] Ma T., Zhang Z., Chen Y. (2021). Delivery of Nitric Oxide in the Cardiovascular System: Implications for Clinical Diagnosis and Therapy. *International Journal of Molecular Sciences*.

[64] Raut M. S., Maheshwari A. (2017). Inhaled Nitric Oxide, Methemoglobinemia, and Route of Delivery. *Saudi Journal of Anaesthesia*.

[65] Taylor M. B., Christian K. G., Patel N., Churchwell K. B. (2001). Methemoglobinemia: Toxicity of Inhaled Nitric Oxide Therapy. *Pediatric Critical Care Medicine*.

[66] Rehman H. U. (2001). Methemoglobinemia. *Western Journal of Medicine*.

[67] Petit P. C., Fine D. H., Vásquez G. B., Gamero L., Slaughter M. S., Dasse K. A. (2017). The Pathophysiology of Nitrogen Dioxide During Inhaled Nitric Oxide Therapy. *ASAIO Journal*.

[68] Gorguner M., Akgun M. (2010). Acute Inhalation Injury. *The Eurasian Journal of Medicine*.

[69] Occupational Safety and Health Administration (2025). Nitric Oxide. https://www.osha.gov/chemicaldata/21.

[70] Centers for Disease Control and Prevention (2014). Nitrogen Dioxide-IDLH. *The National Institute for Occupational Safety and Health*.

[71] Gianni S., Di Fenza R., Araujo Morais C. C. (2022). High-Dose Nitric Oxide from Pressurized Cylinders and Nitric Oxide Produced by an Electric Generator from Air. *Respiratory Care*.

[72] Wiegand S. B., Safaee Fakhr B., Carroll R. W., Zapol W. M., Kacmarek R. M., Berra L. (2020). Rescue Treatment With High-Dose Gaseous Nitric Oxide in Spontaneously Breathing Patients With Severe Coronavirus Disease 2019. *Critical Care Explorations*.

[73] Khan I., Saeed K., Khan I. (2019). Nanoparticles: Properties, Applications and Toxicities. *Arabian Journal of Chemistry*.

[74] Afzal O., Altamimi A. S. A., Nadeem M. S. (2022). Nanoparticles in Drug Delivery: From History to Therapeutic Applications. *Nanomaterials*.

[75] Schairer D., Martinez L. R., Blecher K. (2012). Nitric Oxide Nanoparticles: Pre-Clinical Utility as a Therapeutic for Intramuscular Abscesses. *Virulence*.

[76] Pieretti J. C., Rubilar O., Weller R. B., Tortella G. R., Seabra A. B. (2021). Nitric Oxide (NO) and Nanoparticles-Potential Small Tools for the War Against COVID-19 and Other Human Coronavirus Infections. *Virus Research*.

[77] Williams A. T., Muller C. R., Govender K. (2020). Control of Systemic Inflammation Through Early Nitric Oxide Supplementation With Nitric Oxide Releasing Nanoparticles. *Free Radical Biology and Medicine*.

[78] Shurbaji S., El-Sherbiny I. M., Alser M. (2021). Nitric Oxide Releasing Hydrogel Nanoparticles Decreases Epithelial Cell Injuries Associated With Airway Reopening. *Frontiers in Bioengineering and Biotechnology*.

[79] Wieler L., Vittos O., Mukherjee N., Sarkar S. (2023). Reduction in the COVID-19 Pneumonia Case Fatality Rate by Silver Nanoparticles: a Randomized Case Study. *Heliyon*.

[80] Almanza-Reyes H., Moreno S., Plascencia-López I. (2021). Evaluation of Silver Nanoparticles for the Prevention of SARS-CoV-2 Infection in Health Workers: In Vitro and In Vivo. *PLoS One*.

[81] Rana M. M. (2021). Polymer-Based nano-therapies to Combat COVID-19 Related Respiratory Injury: Progress, Prospects, and Challenges. *Journal of Biomaterials Science, Polymer Edition*.

